# Diffuse Alveolar Hemorrhage Occurring Suddenly in a Diabetic Patient With Asthma Exacerbation

**DOI:** 10.7759/cureus.51893

**Published:** 2024-01-08

**Authors:** Svetoslav Bardarov, Chong Vue, Rochell Santana, Jay Nfonoyim

**Affiliations:** 1 Pathology and Laboratory Medicine, Richmond University Medical Center, Staten Island, USA; 2 Pulmonary and Critical Care, Richmond University Medical Center, Staten Island, USA

**Keywords:** goodpasture's syndrome, poor asthma outcomes, autopsy pathologist, anatomic and clinical pathology, teaching in emergency medicine, pulmonary-renal syndrome, diffuse alveolar hemorrhage

## Abstract

Diffuse alveolar hemorrhage (DAH) is a rare but potentially life-threatening condition characterized by bleeding into the alveolar spaces of the lungs. DAH can occur due to a wide range of etiologies including autoimmune diseases, infections, drugs, and malignancies. The clinical presentation is variable and may include cough, dyspnea, fever, and hemoptysis. Diagnosis is often challenging due to the nonspecific symptoms and a lack of definitive diagnostic criteria. Treatment is primarily aimed at addressing the underlying cause and providing supportive care.

## Introduction

Diffuse alveolar hemorrhage (DAH) is a rare but life-threatening condition characterized by the presence of blood in the alveolar spaces of the lungs, leading to impaired oxygenation and potentially fatal outcomes.

The origin of DAH can be attributed to pulmonary arterioles or venules, and it can present as bilateral diffuse pulmonary involvement or as focal, unilateral findings. The etiologies underlying DAH can be highly variable, and in many times, the identification of the etiology may be challenging [1].

The incidence of DAH is low, and it is typically associated with underlying conditions such as autoimmune diseases, vasculitides, infections, drugs, and malignancies. DAH can also be idiopathic, with no clear underlying cause. The symptoms of DAH include shortness of breath, cough, and hemoptysis.

The morbidity and mortality of DAH are high, with reported mortality rates ranging from 33% to 50% [2] [3]. The prognosis for DAH is largely dependent on the underlying cause and the severity of the condition at the time of diagnosis. Early recognition and appropriate management are crucial for improving outcomes in patients with DAH.

This publication presents the autopsy findings of a 40-year-old diabetic male patient who presented with a deteriorating asthma attack.

## Case presentation

A 40-year-old male with a medical history of asthma, hypertension, type 2 diabetes mellitus, and hyperlipidemia presented to the emergency department with difficulty breathing. Initial assessment revealed an oxygen saturation level of 80%, which improved to 100% with bilevel positive airway pressure (BiPAP). However, subsequent evaluations indicated moderate respiratory depression, accessory muscle use, and elevated PaCO2 on BiPAP, prompting the decision to intubate. The patient was diagnosed with acute hypercapnic/hypoxic respiratory failure secondary to acute asthma exacerbation, uncontrolled hypertension, high glucose levels, and pneumonia, evolving into acute respiratory distress syndrome (ARDS). During the hospital stay, the patient received several medications including insulin, pantoprazole, amlodipine, nicardipine, furosemide, and vancomycin among others. Following initial improvement, the patient experienced a sudden onset of acute hemoptysis on hospital day 17. This progressed rapidly to a massive episode, with aspiration of 900 ml of blood with rapid desaturation. Emergency intubation and resuscitation efforts were instituted; however, the patient expired within one hour. The laboratory values at the time of death were presented in Table 1.

**Table 1:** Laboratory values at the time of death WBC: white blood cell; RBC: red blood cell; Na: sodium; K: potassium; Cl: chlorine; BUN: blood urea nitrogen; Neg: negative; Pos: positive

Hematology			Chemistry			Urine		
Analyte	Value	Range	Analyte	Value	Range	Analyte	Value	Range
WBC	18	4.0-11.2 k/ul	Na	145	136-145 mmol/l	Glucose	100	Neg
RBC	3.32	4.0-11.2 m/ul	K	4.8	3.5-5.5 mmol/l	Ketones	Neg	Neg
Hemoglobin	8.8	13.7-17.5 g/dl	Cl	27	98-107 mmol/l	Blood	Pos	Neg
Platelets	330	150-400 k/ul	BUN	50	7-18 mg/dl	pH	7.0	
Neutrophils	82	30-70%	Creatinine	1.94	0.55-1.02 mg/dl	Protein	300	Neg
Lymphocytes	10	23-54%	Glucose	217	74-106 mg/dl			

**Figure 1:**
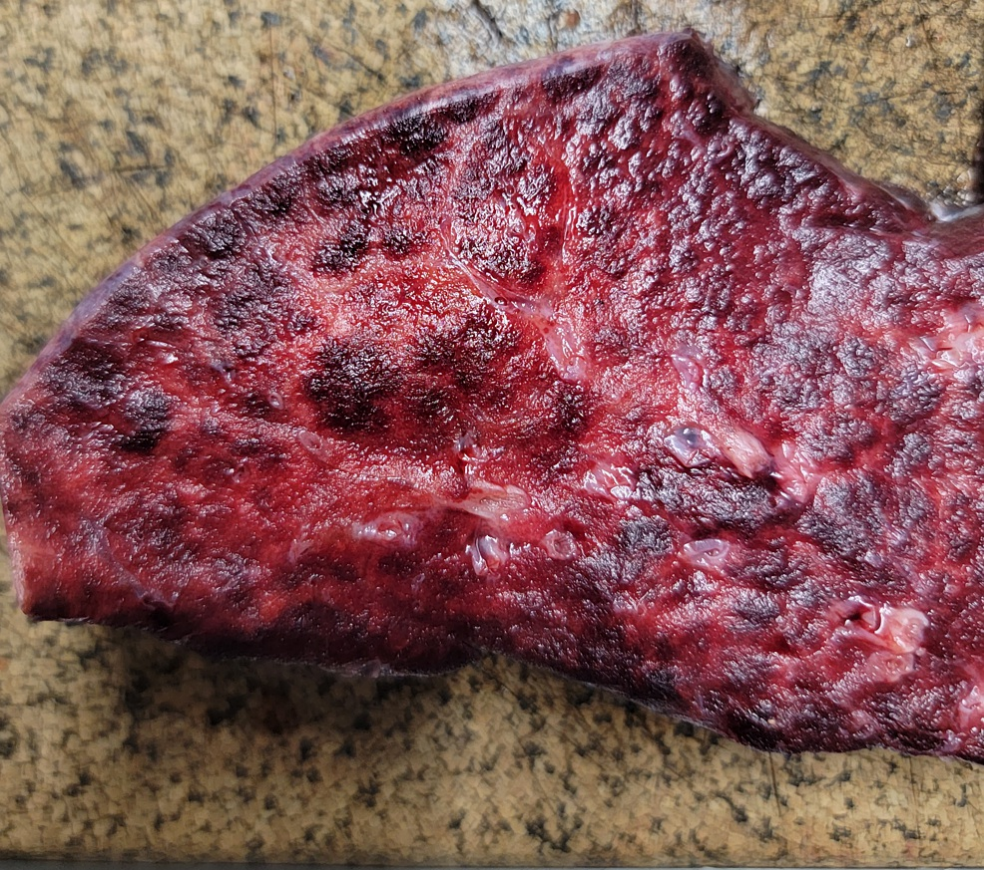
Right lung, middle lobe showing a marked consolidation

The gross pathological examination demonstrated marked bilateral lung consolidation (left lung: 800 g; right lung: 1000 g) with no evidence of pleural, pericardial, or peritoneal effusion (Figure 1).

Additionally, there was no evidence of an acute cerebrovascular accident. Grossly, the heart weighed 470 grams and exhibited a focus of myocardial fibrosis (~1.1 cm) involving the anterior myocardium; however, the coronary arteries did not exhibit any significant evidence of advanced atherosclerosis.

**Figure 2:**
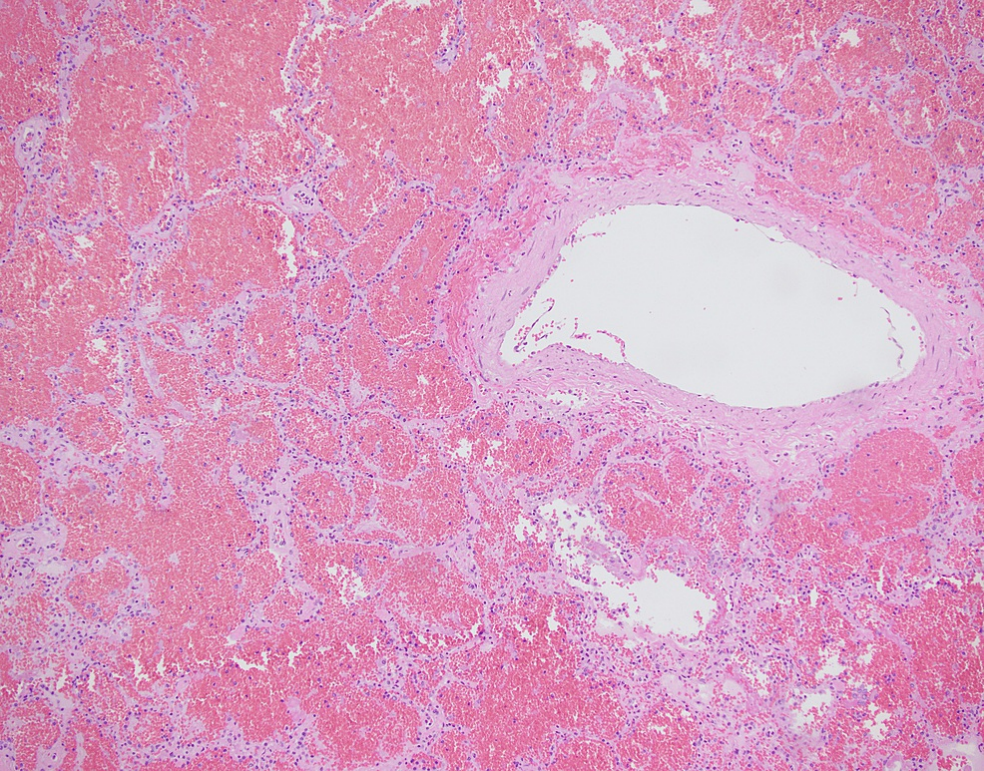
Right lung, middle lobe: intra-alveolar hemorrhage (200x, H&E)

**Figure 3:**
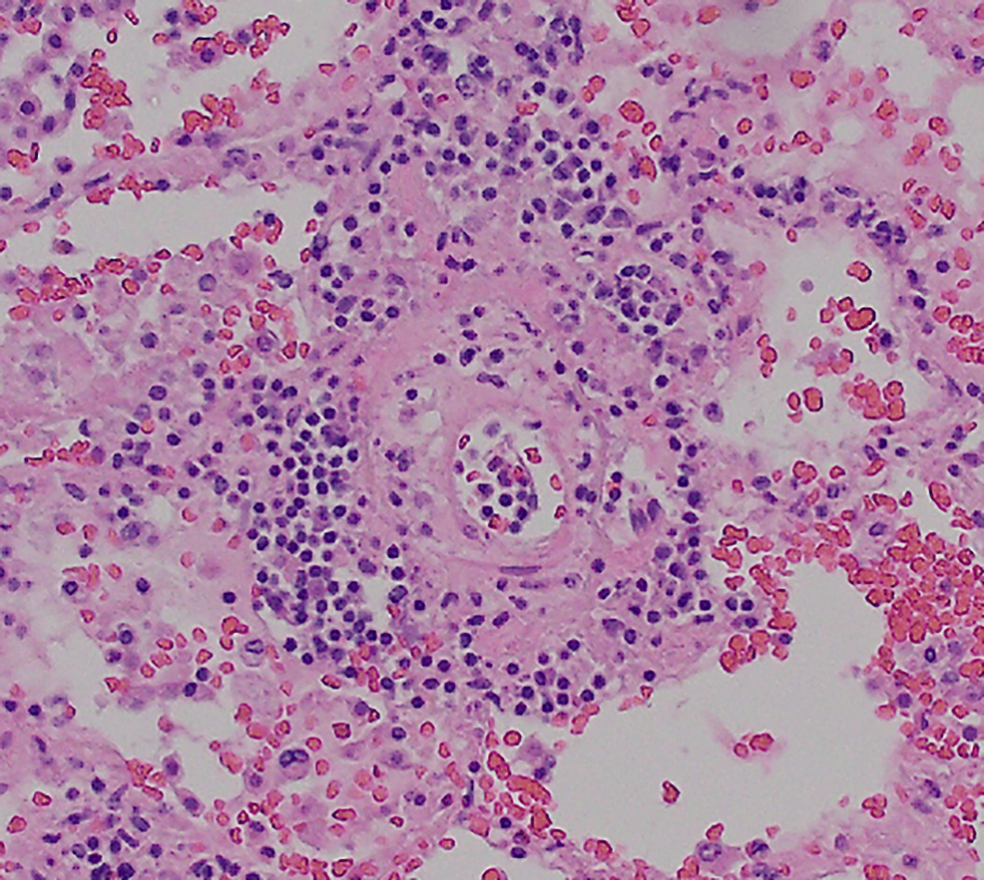
Right lung, middle lobe showing a perivascular lymphoplasmacytic infiltration (400x, H&E)

**Figure 4:**
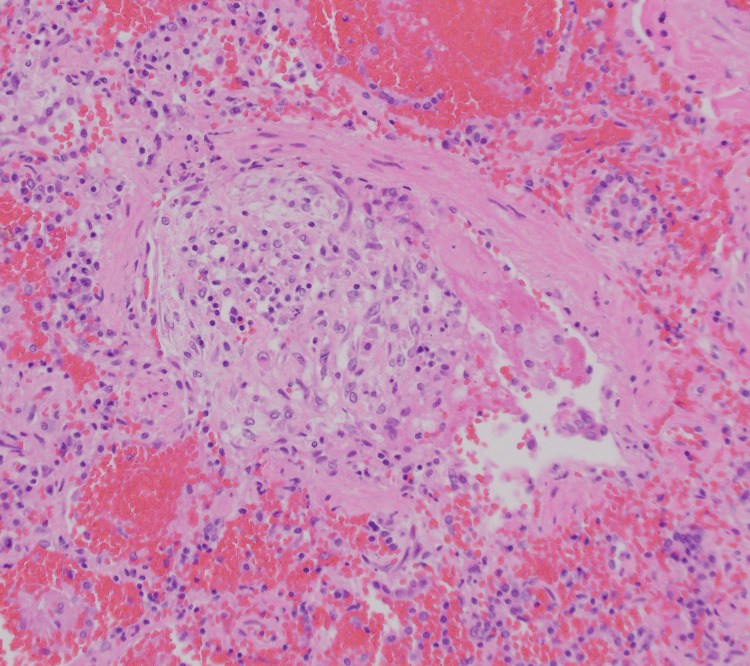
Right lung, middle lobe: microvascular thrombosis (400x, H&E)

The microscopic examination of both lungs displayed diffuse bilateral pulmonary intra-alveolar hemorrhage with rare arteries showing perivascular lymphoplasmacytic infiltrates and rare organizing microemboli with no evidence of fibrinoid necrosis (Figure 2, Figure 3, and Figure 4). A focal neutrophilic peribronchial infiltration in the left lower lobe was identified with no evidence of diffuse alveolar damage.

**Figure 5:**
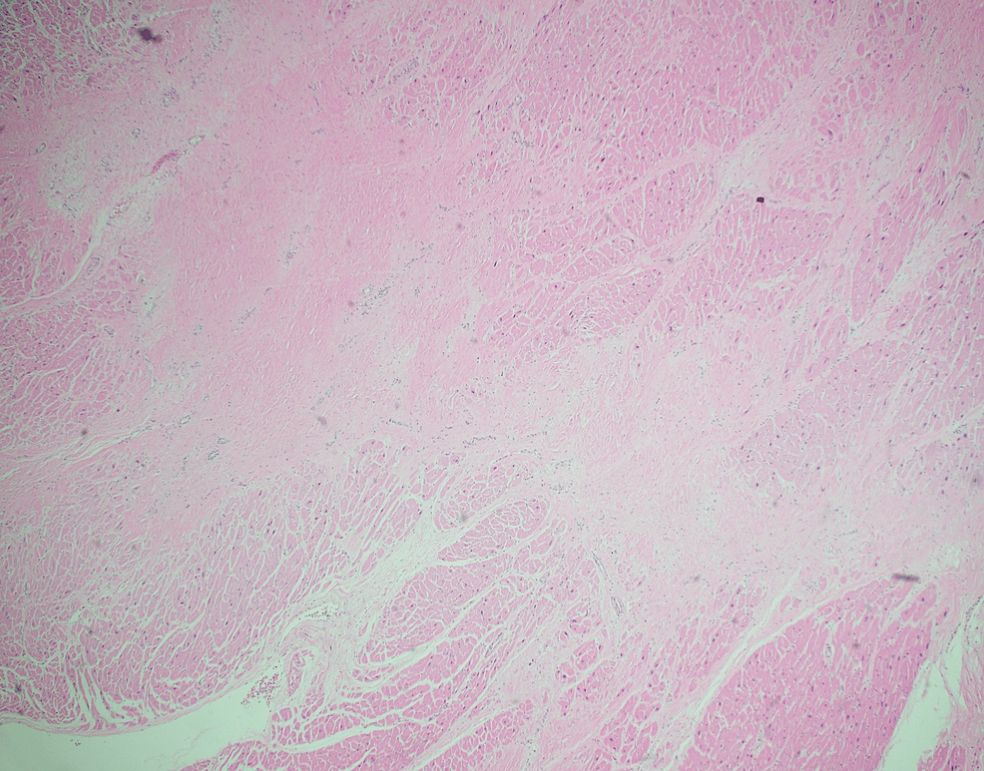
Left myocardium showing area of fibrosis consistent with past myocardial infarction (4x, H&E)

Furthermore, the microscopic examination of the heart revealed healed myocardial infarction of approximately 1.1 cm, with no advanced atherosclerotic changes or capillaritis in the cardiac vessels and no evidence of myocarditis (Figure 5).

**Figure 6:**
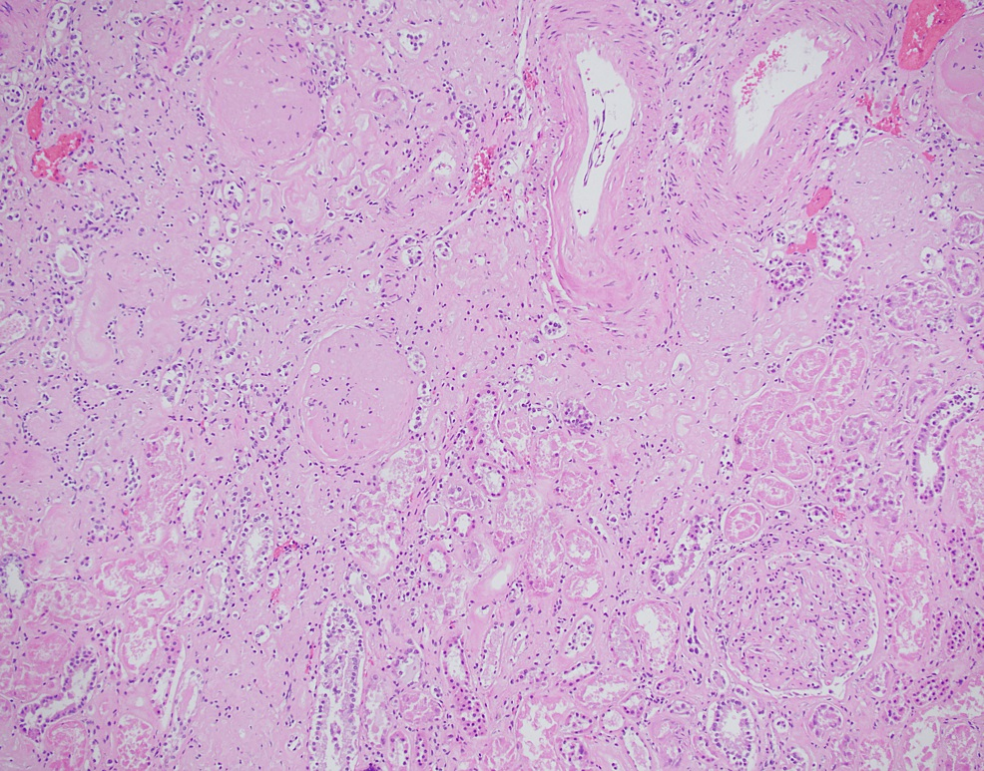
Right kidney showing glomerulosclerosis, tubular atrophy, and chronic interstitial nephritis (200x, H&E)

The microscopic evaluation of the kidneys showed advanced diabetic nephropathy with areas of segmental sclerosis and hyaline insudation, glomerulosclerosis, moderate arteriosclerosis, significant interstitial fibrosis, tubular atrophy, and chronic interstitial nephritis. There was no evidence of crescentic glomerulonephritis (Figure 6).

## Discussion

DAH is a rare but serious complication of various autoimmune and vasculitic diseases. Pulmonary-renal syndrome (PRS) is a condition characterized by the presence of both DAH and glomerulonephritis, which can occur together in various autoimmune and vasculitic diseases such as systemic lupus erythematosus, microscopic polyangiitis, and Goodpasture's syndrome. DAH is an important feature of PRS and can lead to significant morbidity and mortality. Early diagnosis and treatment of PRS are critical in improving disease outcomes.

DAH can be classified using several systems, but the most commonly used is based on etiology. DAH can be divided into primary and secondary types. Primary DAH is caused by autoimmune diseases such as microscopic polyangiitis and systemic lupus erythematosus, while secondary DAH can result from infections, drugs, and malignancies [4,5]. If no etiology can be identified, the DAH is classified as bland DAH.

In chest radiography, the presence of either patchy or diffuse alveolar opacities may be observed. Repeated instances of hemorrhage can result in the development of reticular interstitial opacities, indicative of pulmonary fibrosis, typically characterized by minimal honeycombing. Computed tomography scans may reveal regions of consolidation interspersed with areas exhibiting ground-glass attenuation, alongside preserved normal lung areas. These observed radiological manifestations generally lack specificity [6].

Clues to specific diagnosis frequently include exposure to possible offending drugs or other causative agents including crack cocaine which can cause capillaritis, pre-existing infections with severe acute respiratory syndrome coronavirus 2 (SARS-CoV-2) with ARDS and microvascular thrombosis, and pre-existing conditions like rheumatoid arthritis or systemic vasculitis.

When the diagnosis of DAH is suspected, a laboratory workup should include antineutrophil cytoplasmic antibodies (ANCA), anti-glomerular basement membrane antibodies (anti-GBM), antiphospholipid antibodies, drug screening, and bronchoalveolar lavage. If the etiology remains unidentified following an exhaustive clinical assessment encompassing imaging studies, serologic investigations, and bronchoscopy, contemplation of surgical biopsy is warranted. The selection of the organ for biopsy should be guided by the degree of suspicion pertaining to a particular causative factor [6].

In this particular case, it is difficult to ascertain the underlying cause of the pulmonary alveolar hemorrhage with absolute certainty. Despite being a non-smoker and not having a history of vaping or illicit drug use, the patient's medical records indicate that they were compliant with their prescribed medications. However, it is worth noting that the patient was diagnosed with hypertension and diabetes, but had not undergone a kidney biopsy to evaluate the grade of the diabetic nephropathy. Additionally, the patient had not undergone any auto-antibody workup. Based on these factors as well as the pathologic findings, the diagnosis of PRS as the cause of death cannot be established with certainty.

After a thorough examination, the final diagnosis for the patient is an acute asthma exacerbation caused by bronchopneumonia in the left lower lobe. This was followed by a rapidly evolving complication of diffuse bilateral bland alveolar hemorrhage. These conditions emerged against a background of advanced diabetic nephropathy, hypertension, and a history of myocardial infarction.

## Conclusions

This case highlights the diagnostic challenge of DAH, a rare but life-threatening condition. Although an exact cause could not be pinpointed, the collaborative efforts of the clinical and pathology teams significantly advanced the understanding of the progression of the patient's illness.
